# Epidural analgesia does not impact recurrence or mortality in patients after rectal cancer resection

**DOI:** 10.1038/s41598-020-79657-5

**Published:** 2021-01-13

**Authors:** Hsiang-Ling Wu, Ying-Hsuan Tai, Shih-Pin Lin, Shung-Haur Yang, Mei-Yung Tsou, Kuang-Yi Chang

**Affiliations:** 1 Department of Anaesthesiology, Taipei Veterans General Hospital, No. 201, Sec. 2, Shih-pai Rd., Taipei, 11217 Taiwan; 2 School of Medicine, National Yang-Ming University, Taipei, Taiwan; 3 Department of Anaesthesiology, Shuang Ho Hospital, Taipei Medical University, New Taipei City, Taiwan; 4 Department of Anaesthesiology, School of Medicine, College of Medicine, Taipei Medical University, Taipei, Taiwan; 5 Division of Colon and Rectal Surgery, Department of Surgery, Taipei Veterans General Hospital, Taipei, Taiwan; 6 National Yang-Ming University Hospital, Yilan, Taiwan

**Keywords:** Gastroenterology, Oncology, Risk factors

## Abstract

The relationship between epidural analgesia and rectal cancer outcome is not fully clarified. We aimed to investigate the putative effect of epidural analgesia on the risks of recurrence and mortality after rectal tumour resection. In this monocentric cohort study, we consecutively enrolled patients with stage I–III rectal cancer who underwent tumour resection from 2005 to 2014. Patients received epidural analgesia or intravenous opioid-based analgesia for postoperative pain control. Primary endpoint was first cancer recurrence. Secondary endpoints were all-cause mortality and cancer-specific mortality. We collected 1282 patients in the inverse probability of treatment weighting analyses, and 237 (18.5%) used epidurals. Follow-up interval was median 46.1 months. Weighted Cox regression analysis showed the association between epidural analgesia and recurrence-free survival was non-significant (adjusted hazard ratio [HR] 0.941, 95% CI 0.791–1.119, *p* = 0.491). Similarly, the association between epidural analgesia and overall survival (HR 0.997, 95% CI 0.775–1.283, *p* = 0.984) or cancer-specific survival (HR 1.113, 95% CI 0.826–1.501, *p* = 0.482) was non-significant either. For sensitivity tests, quintile stratification and stepwise forward model selection analyses showed similar results. We did not find a significant association between epidural analgesia and risk of recurrence, all-cause mortality, or cancer-specific mortality in patients with rectal cancer undergoing tumour resection.

## Introduction

Rectal cancer is ranked as the seventh most common cancer worldwide, resulting in approximately 700,000 new cases and 310,000 cancer death in 2018^1^. While surgery is an important treatment modality for non-metastatic rectal cancer, tumour resection may produce a microenvironment favorable for the development of residual cancer cells, facilitate tumour spread into the circulatory and lymphatic systems, and allow for subsequent metastatic disease^2,3^. There is accumulating evidence that regional anaesthesia and analgesia could modify the process of tumourigenesis and protect against the risk of cancer recurrence after tumour resction^2,3^. A recent study showed epidural anaesthesia and analgesia (EA) reduces the production of inflammatory cytokines and stress hormones in patients undergoing radical tumour resection for colon cancer^4^. Accordingly, it has been hypothesised that perioperative use of EA may reduce the risk of cancer recurrence after tumour resection through alleviating patient’s stress response and inflammation process^2–4^.

Retrospective studies have also demonstrated perioperative use of EA was associated with longer survival after curative surgery for colorectal cancer^5–9^. However, the relationship between EA and rectal cancer outcome is not completely understood because of conflicting findings and several study limitations, including small patient samples (< 1000 subjects)^5,6,8–11^ and mixed groups of patients with colon and rectal cancer^6–8,10^. Rectal cancer is distinct from colon cancer in stage-dependent treatment strategies^12^, profiles of gene mutations^13^, surgical complication rate and short-term outcome^14^, and long-term survival^15^. Therefore, the two tumour entities should be investigated separately.

The objective of this study was to evaluate the relationship between EA and long-term risks of recurrence and mortality after surgical resection of primary rectal cancer. We also aimed to identify potential predictors for postoperative recurrence and mortality of rectal cancer. Specifically, we hypothesised that EA was associated with lower risks of cancer recurrence, all-cause mortality, and cancer-specific mortality after rectal cancer resection.

## Methods

**Figure 1:**
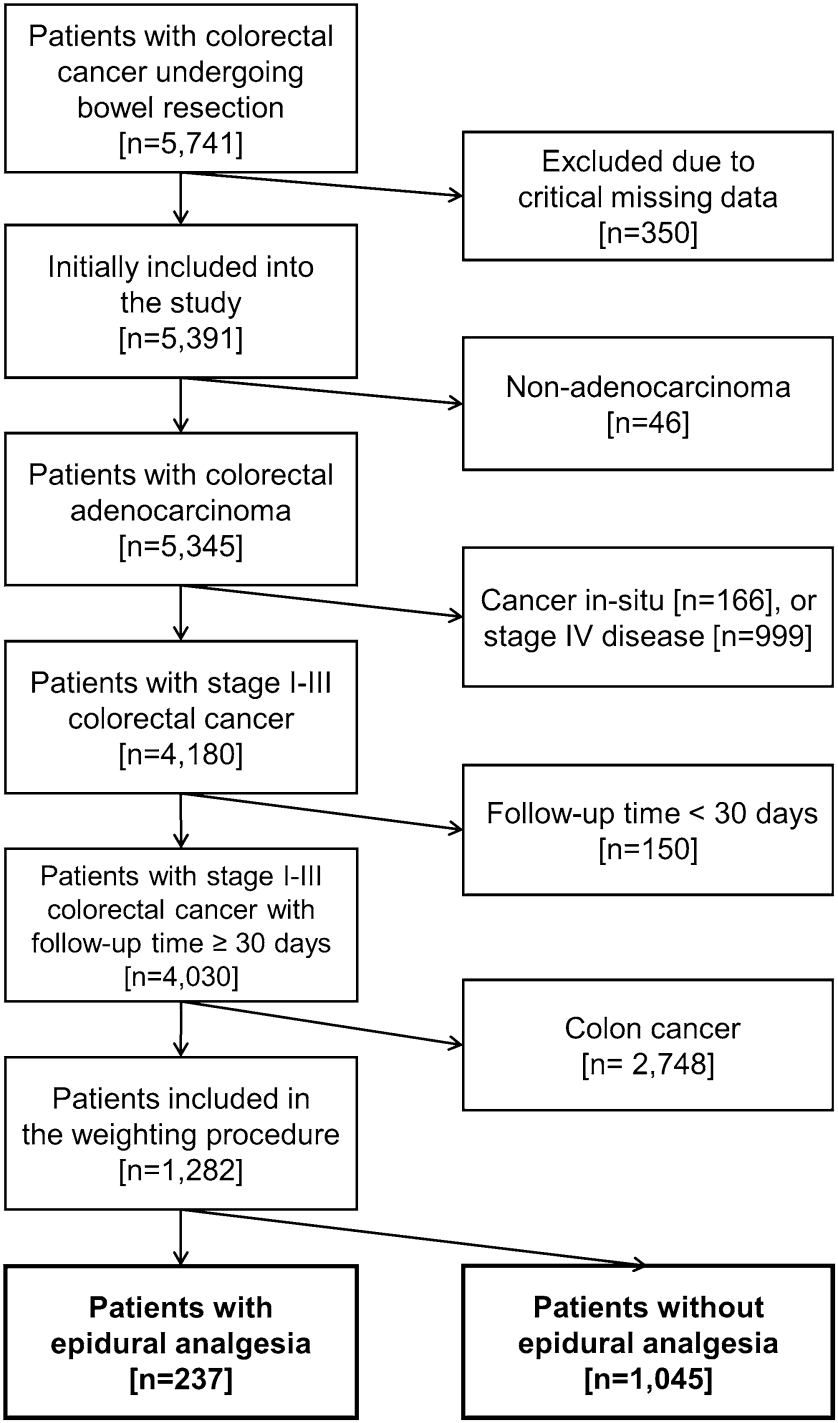
Flow diagram for patient selection.

We obtained the ethics approval from the Institutional Review Board (IRB), Taipei Veterans General Hospital (IRB-TPEVGH No. 2015-11-010CC) in Taiwan. Written informed consent was waived by the IRB, and the data were de-identified before analysis. All methods were conducted in accordance with the relevant guidelines and regulations. We reviewed the electronic medical records of the medical centre and consecutively enrolled patients diagnosed with colon or rectal cancer. Inclusion criteria were age ≥ 20 years and patients undergoing bowel resection for primary tumours located at colon or rectum at the centre from 2005 to 2014. We excluded patients with missing data, follow-up interval < 30 days, histology-proven non-adenocarcinoma, carcinoma in-situ, distant organ metastasis, and colon cancer. We defined rectal cancer as the tumour located within 15 cm of the anal verge measured by colonoscopy. Patients were further divided into two groups, EA group and non-EA group, based on whether the general anaesthesia for surgical resection of rectal cancer was combined with a successful EA, which was defined as the epidural covering the area of surgical incision and site of pain without causing unnecessary motor blockade (Fig. 1).

### Protocol of pain control

For major abdominal surgery, EA is one of the analgesic strategies to manage postoperative pain at this centre^16,17^. Patients receiving EA would undergo the placement of epidural catheter at the level of T10 to T12 spine and the position and function of epidural catheters were tested with 2–3 mL of 2% lidocaine one day before the surgery. EA was typically initiated with a bolus of lidocaine 60–100 mg prior to surgical incision and a subsequent continuous infusion of bupivacaine 0.25% or 0.5% with or without fentanyl 1 to 2 µg·mL^−1^ at a rate of 5–10 mL h^−1^. After surgery, EA was used for 3 days to treat post-surgery pain. Several reasons precluded patients from EA, including contraindications to epidural insertion (e.g. preoperative platelet count < 100,000 cells μL^−1^ or international normalized ratio > 1.3), epidurals not working, and patient’s preferences. For patients without EA, we offered intravenous as-needed or patient-controlled opioid-based analgesia^18,19^.

### Determination of recurrence and death

An anaesthesiologist (Y.H.T.) who was blinded to group allocation made an independent assessment of cancer outcome. Primary outcome is recurrence-free survival (RFS), defined as the interval between the time of surgery and the first cancer recurrence. We determined the recurrence based on the localized or distant metastatic deposits detected by imaging studies, including plain film, computerized tomography scan, and magnetic resonance imaging. Tissue biopsy was performed to confirm the presence of recurrent cancer if possible. Secondary outcome is overall survival (OS), defined as the interval between the time of surgery and death from all causes, and cancer-specific survival (CSS), defined as the interval between the time of surgery and death from rectal cancer. The date of death was derived from medical records and death certificates. Survival times were the corresponding censored observations in patients without recurrence or death. Patient’s status was followed up until August 31, 2016.

### Clinical and pathologic covariates

We used the electronic medical database to collect potential confounding factors for rectal cancer outcome. Clinical covariates were demographics, American Society of Anesthesiologists (ASA) class, coexisting diseases, pretreatment level of carcinoembryonic antigen (CEA)^20^, open or laparoscopic surgery, anaesthesia time, perioperative transfusion of red blood cells (during or within 7 days after surgery)^21–23^, and preoperative or postoperative chemotherapy or radiotherapy (within 90 days before or after surgery). ASA classes were dichotomised into ≥ 3 and < 3 due to their obvious difference in postoperative mortality and surgical risk^24^. Anaesthesia time was defined as the interval from the induction of anaesthesia to extubation of endotracheal tubes. At this centre, adjuvant chemotherapy for rectal cancer was typically composed of leucovori and oxaliplatin or 5-fluorouracil, capecitabine, or tegafur-uracil^25^. The adjuvant chemotherapy and radiotherapy were based on patients’ cancer stage, pathologic features, general conditions, and preferences. Important pathologic characteristics included American Joint Committee on Cancer (AJCC) cancer stage^26^, tumour distance from the anal verge^27^, differentiation grade^28^, mucinous and signet-ring subtypes^29^, lymphovascular and perineural invasion^30,31^.

### Statistical analysis

We used Shapiro–Wilk test and Kolmogorov–Smirnov test as a measure of normality. For non-normal continuous variables, logarithmic transformation was used to reduce skewness in the statistical analyses. We applied the inverse probability of treatment weighting (IPTW) model for propensity score to detect pseudo-populations and reduce the imbalanced distribution of collected covariates as follows^32^. First, logistic regression analysis was conducted to estimate the probability of undergoing EA based on a list of patient characteristics, i.e. propensity score. (Supplementary Table S1) Cox regression analysis was then weighted with the inverse of propensity score with a truncation at 99% to diminish the impact of large weights. We used standardised differences to evaluate the balance of covariates between groups for the original and weighted samples. We used weighted Cox regression analyses to evaluate the association of EA with RFS, OS, or CSS. To increase the robustness of our results, propensity score stratification and stepwise forward model selection analyses were implemented as sensitivity tests. In propensity score stratification, we used the quintiles of propensity scores to classify the patients into five equal-size groups and conducted stratified Cox regression analysis to obtain a pooled hazard ratio. We used multivariable Cox regression analysis with stepwise forward model selection and the entry and removal significance criteria of 0.1 and 0.05 to determine the independent factors associated with RFS, OS, and CSS. We considered *p* < 0.05 statistically significant. All the statistical analyses were performed using SAS software, version 9.4 (SAS Institute Inc., Cary, NC, USA).

## Results

After meeting the selection criteria, we included a total of 1282 patients, 237 (18.5%) of whom underwent EA. Follow-up interval was median 46.1 months (interquartile range 24.3–73.5). Patients with EA were older and more likely to undergo open surgery with longer anaesthesia time compared to non-EA group. There was no significant imbalance in pathologic characteristics between groups. The IPTW procedure created a pseudo-population of 1073 subjects in EA group and 1181 in non-EA group. The imbalance of covariate distribution was substantially reduced by IPTW (Table 1).

**Table 1:** Demographic, clinical, and pathologic characteristics of included patients before and after IPTW.

	Original		SDD	After IPTW		SDD
	EA (N = 237)	Non-EA (N = 1045)		EA (N = 1073)	Non-EA (N = 1181)	
Age, year	69 ± 13	66 ± 13	20.2	67 ± 13	66 ± 13	5.1
Sex, male	91 (38.4%)	409 (39.1%)	1.5	438 (40.8%)	471 (39.9%)	1.9
ASA class ≥ 3	72 (30.4%)	306 (29.3%)	2.4	312 (29.1%)	351 (29.7%)	1.3
Body mass index, kg m^−2^	23.7 ± 3.6	24.1 ± 3.9	10.0	23.7 ± 3.5	24.1 ± 3.9	10.9
**Comorbidities**						
Diabetes	51 (21.5%)	206 (19.7%)	4.5	211 (19.6%)	234 (19.8%)	0.5
Coronary artery disease	23 (9.7%)	88 (8.4%)	4.5	102 (9.5%)	106 (8.9%)	2.0
Heart failure	9 (3.8%)	55 (5.3%)	7.1	57 (5.4%)	62 (5.3%)	0.3
Stroke	15 (6.3%)	63 (6.0%)	1.2	66 (6.2%)	70 (5.9%)	1.3
Chronic kidney disease	33 (13.9%)	124 (11.9%)	6.1	142 (13.2%)	141 (11.9%)	3.9
Pretreatment CEA, μg L^−1^	2.7 (2.1–4.5)	2.7 (2.0–5.0)	2.3	2.6 (2.1–3.9)	2.7 (2.0–4.9)	2.0
Laparoscopic surgery	2 (0.8%)	74 (7.1%)	32.4	20 (1.9%)	75 (6.4%)	22.5
Anaesthesia time, min	285 (240–330)	300 (255–375)	39.1	300 (240–345)	300 (245–375)	10.3
pRBC transfusion	55 (23.2%)	226 (21.6%)	3.8	229 (21.3%)	249 (21.1%)	0.6
Preoperative C/T ± R/T	51 (21.5%)	273 (26.1%)	10.8	244 (22.7%)	286 (24.2%)	3.6
Postoperative C/T	86 (36.3%)	480 (45.9%)	19.7	446 (41.5%)	525 (44.5%)	5.9
Postoperative R/T	8 (3.4%)	40 (3.8%)	2.4	33 (3.1%)	43 (3.7%)	3.0
**Pathologic features**						
Cancer stage			0.2			1.0
I	72 (30.4%)	311 (29.8%)		335 (31.2%)	363 (30.7%)	
II	80 (33.8%)	367 (35.1%)		373 (34.7%)	410 (34.7%)	
III	85 (35.9%)	367 (35.1%)		365 (34.0%)	408 (34.6%)	
Distance from anal verge, cm	8.0 ± 3.4	8.2 ± 3.7	4.3	8.0 ± 3.5	8.2 ± 3.7	5.4
Tumour differentiation			3.1			1.8
Good	20 (8.7%)	62 (6.1%)		81 (7.5%)	77 (6.5%)	
Moderate	200 (86.6%)	915 (89.9%)		947 (88.2%)	1056 (89.4%)	
Poor	11 (4.8%)	41 (4.0%)		45 (4.2%)	49 (4.1%)	
Mucinous histology	7 (3.0%)	28 (2.8%)	1.7	33 (3.1%)	34 (2.8%)	1.3
Signet-ring histology	3 (1.3%)	23 (2.3%)	7.3	28 (2.6%)	26 (2.2%)	2.5
Lymphovascular invasion	32 (13.9%)	163 (16.0%)	5.9	161 (15.0%)	187 (15.8%)	2.2
Perineural invasion	10 (4.3%)	80 (7.9%)	14.8	64 (5.9%)	85 (7.2%)	5.0

Values were mean ± SD, counts (percent), or median (interquartile range).

*SDD* standardised difference, *ASA* American Society of Anesthesiologists, *CEA* carcinoembryonic antigen, *C/T* chemotherapy, *EA* epidural analgesia, *IPTW* inverse probability of treatment weighting, *pRBC* packed red blood cells, *R/T* radiotherapy.

### Epidural analgesia and cancer recurrence

**Figure 2:**
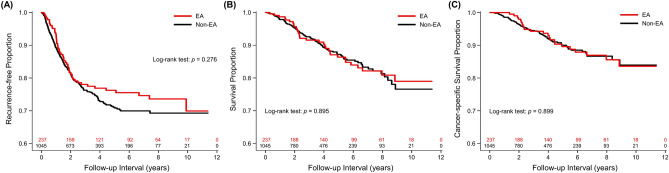
Kaplan–Meier curves for (**A**) recurrence-free survival, (**B**) overall survival, and (**C**) cancer-specific survival of epidural and non-epidural groups with number of subjects at risk.

The 3-year and 5-year recurrence-free survival rates were 78.0% (95% confidence interval [CI] 72.3–83.7) and 76.2% (95% CI 70.3–82.1) in EA group and 76.3% (95% CI 73.6–79.0) and 71.1% (95% CI 68.0–74.2) in non-EA group, respectively. EA was not associated with RFS in the univariate analysis (hazard ratio [HR] 0.848, 95% CI 0.630–1.141, *p* = 0.277). (Fig. 2A) After the effects of covariates were controlled, the association between EA and RFS remained non-significant, in the IPTW analysis (adjusted HR 0.941, 95% CI 0.791–1.119, *p* = 0.491), quintile stratification (adjusted HR 0.998, 95% CI 0.719–1.383, *p* = 0.988), and forward model selection (adjusted HR 0.964, 95% CI 0.702–1.324, *p* = 0.821). Forward model selection identified seven independent predictors for RFS, including pretreatment CEA, tumour distance from anal verge, cancer stage, lymphovascular invasion, perineural invasion, preoperative chemotherapy and/or radiotherapy, and postoperative radiotherapy (Table 2).

**Table 2:** Forward model selection for recurrence-free survival.

	HR (95% CI)	*p*
Epidural analgesia	0.964 (0.702–1.324)	0.821
Pretreatment CEA, μg L^−1a^	1.524 (1.183–1.964)	0.001
Distance from anal verge, cm^b^	0.949 (0.915–0.985)	0.005
**Cancer stage**		< 0.001
II vs. I	2.119 (1.415–3.175)	< 0.001
III vs. I	3.416 (2.288–5.101)	< 0.001
Lymphovascular invasion	1.605 (1.177–2.187)	0.003
Perineural invasion	1.787 (1.239–2.578)	0.002
Preoperative C/T ± R/T	1.786 (1.356–2.351)	< 0.001
Postoperative R/T	2.248 (1.419–3.562)	0.001

*HR* hazard ratio, *CI* confidence interval, *CEA* carcinoembryonic antigen, *C/T* chemotherapy, *R/T* radiotherapy.

^a^On base-10 logarithmic scale.

^b^On base-2 logarithmic scale.

### Epidural analgesia and all-cause mortality

The 3-year and 5-year overall survival rates were 91.6% (95% CI 87.7–95.5) and 86.4% (95% CI 81.3–91.5) in EA group and 92.6% (95% CI 90.8–94.4) and 87.2% (95% CI 84.7–89.7) in non-EA group, respectively. In the univariate analysis, EA was not associated with OS (HR 0.974, 95% CI 0.655–1.448, *p* = 0.895) (Fig. 2B). After the IPTW adjustment, the association between EA and OS remained non-significant (HR 0.997, 95% CI 0.775–1.283, *p* = 0.984). Sensitivity analyses showed similar results, including quintile stratification (HR 1.042, 95% CI 0.663–1.636, *p* = 0.858) and forward model selection (HR 1.020, 95% CI 0.668–1.559, *p* = 0.927). Forward model selection determined seven independent risk factors for all-cause mortality, including older age, chronic kidney disease, higher pretreatment CEA level, red cell transfusion, advanced cancer stage, lymphovascular invasion, and preoperative chemotherapy and/or radiotherapy (Table 3).

**Table 3:** Forward model selection for overall survival.

	HR (95% CI)	*p*
Epidural analgesia	1.020 (0.668–1.559)	0.927
Age, year	1.034 (1.017–1.051)	< 0.001
Chronic kidney disease	1.784 (1.124–2.830)	0.014
Pretreatment CEA, μg L^−1a^	1.826 (1.278–2.609)	0.001
pRBC transfusion	1.936 (1.317–2.846)	0.001
**Cancer stage**		< 0.001
II vs. I	1.379 (0.755–2.520)	0.296
III vs. I	2.879 (1.616–5.127)	< 0.001
Lymphovascular invasion	1.778 (1.144–2.766)	0.011
Preoperative C/T ± R/T	2.176 (1.480–3.201)	< 0.001

*HR* hazard ratio, *CI* confidence interval, *CEA* carcinoembryonic antigen, *C/T* chemotherapy, *pRBC* packed red blood cells, *R/T* radiotherapy.

^a^On base-10 logarithmic scale.

### Epidural analgesia and cancer‐specific mortality

The 3-year and 5-year cancer-specific survival rates were 94.3% (95% CI 91.0–97.6) and 89.6% (95% CI 84.9–94.3) in EA group and 94.5% (95% CI 92.9–96.1) and 90.0% (95% CI 87.6–92.4) in non-EA group, respectively. In the univariate analysis, EA was not associated with CSS (HR 0.970, 95% CI 0.608–1.548, *p* = 0.900) (Fig. 2C). After the IPTW adjustment, the association between EA and CSS remained non-significant (HR 1.113, 95% CI 0.826–1.501, *p* = 0.482). Sensitivity analyses showed similar results, including quintile stratification (HR 1.230, 95% CI 0.724–2.089, *p* = 0.444) and forward model selection (HR 0.976, 95% CI 0.548–1.738, *p* = 0.934) (Table 4).

**Table 4:** Forward model selection for cancer-specific survival.

	HR (95% CI)	*p*
Epidural analgesia	0.976 (0.548–1.738)	0.934
ASA class ≥ 3	1.836 (1.117–3.018)	0.017
BMI, kg m^−2^	0.916 (0.864–0.970)	0.003
Pretreatment CEA, μg L^−1a^	2.095 (1.381–3.179)	0.001
pRBC transfusion	1.734 (1.064–2.825)	0.027
**Cancer stage**		< 0.001
II vs. I	1.702 (0.704–4.115)	0.238
III vs. I	4.782 (2.104–10.870)	< 0.001
Perineural invasion	2.375 (1.296–4.354)	0.005
Preoperative C/T ± R/T	3.164 (2.010–4.981)	< 0.001

*HR* hazard ratio, *CI* confidence interval, *ASA* American Society of Anesthesiologists, *BMI* body mass index, *CEA* carcinoembryonic antigen, *C/T* chemotherapy, *pRBC* packed red blood cells, *R/T* radiotherapy.

^a^On base-10 logarithmic scale.

## Discussion

In this study, our results did not support a definite relationship between perioperative epidural analgesia and postoperative cancer recurrence, all-cause mortality, or cancer-specific mortality in rectal cancer. In contrast to previous studies^5–9^, we found no evidence for the hypothetical benefits of epidural analgesia in cancer control or patient survival after rectal tumour resection. Our study has several strengths to elucidate the relationship of EA with rectal cancer outcome. First, we collected a comprehensive list of clinical and pathologic variables and conducted three types of analytical models to obtain robust results. Second, we investigated rectal cancer as an individual tumour entity, which was lack in previous studies^5–11^. Our highly homogenous cohort increased the internal validity of the observed results and contributed to the current literature regarding the role of regional anaesthesia in cancer surgery.

Prior studies have shown the addition of epidural analgesia to general anaesthesia was associated with better postoperative survival in patients undergoing colorectal cancer resection, but recurrence risk did not differ between epidural and non-epidural groups^5–10^. In contrast to the previous studies, Wurster et al. recently reported no association between EA and risk of recurrence or mortality in colon cancer after propensity score matching analyses^11^. We used propensity score methodology and found similar results. From the clinical perspective, the administration of epidural analgesia is highly dependent on patients’ general condition. Severe weakness or coagulopathy may contraindicate the placement of epidural catheters. This causes an indication bias. To minimise potential confounding effects of covariates, propensity score weighting and matching are powerful tools to cope with the biased distribution of patient characteristics with regard to the use of EA^32^. In a recent multicentre randomised trial, Sessler et al. reported that paravertebral blocks with propofol-based general anaesthesia did not reduce postoperative cancer recurrence compared to opioid analgesia with sevoflurane-based anaesthesia in breast cancer^33^. For rectal or colon cancer, we await the high-quality evidence from the ongoing clinical trials (ClinicalTrials.gov Identifier: NCT01318161 and NCT03700411).

Some limitations are inherent in this retrospective study. First, unrecorded variables cannot be further controlled in the analytical models, including perioperative uses of opioids, tumour-involved margin, genetic profiles of tumour, detailed information of chemotherapy and radiotherapy, and postoperative complication events^34,35^. Of note, our prior study demonstrated no definite correlation between postoperative morphine dose and oncological outcome in patients with colorectal cancer^36^. Second, we did not include data about contraindications to epidurals, such as coagulation profiles and physical frailty. However, this should make the direction of bias away from the null and therefore did not change the conclusion. Third, we did not consider the medical conditions which were intrinsically linked to cancer recurrence and mortality, such as inflammatory bowel disease and concurrent malignancy. Fourth, we did not routinely measure inflammation-related markers (such as lymphocyte count and c-reactive protein) in this cohort and therefore could not adjust for them in the analyses^37^. Fifth, the follow-up period of this study is limited to August 2016. However, the Kaplan–Meier curve for RFS (Table 2A) shows that most of recurrences occurred within 36 months after surgery. Therefore, the follow-up interval of median 46.1 months should be sufficient to evaluate the association of EA with recurrence risk. Finally, the property of the single-centre study and homogeneous sample could also be a disadvantage for the generalizability of our results, especially for hospitals with different clinical settings.

In conclusion, our results did not support the hypothetical beneficial effect of epidural analgesia on postoperative cancer outcomes and survival in rectal cancer. Current indications of epidurals should not be changed in the setting of cancer surgery.

## Supplementary Information

Supplementary Information.

## Data Availability

Due to ethical restrictions, the data cannot be made publicly accessible but are available upon reasonable request. The request should be directed to Dr. Chang (kychang@vghtpe.gov.tw).
