# Supplementary material for: Epidural analgesia does not impact recurrence or mortality in patients after rectal cancer resection
Source: Sci Rep. 2021 Jan 13;11:913. doi: 10.1038/s41598-020-79657-5 (PMC7807023; doi:10.1038/s41598-020-79657-5)
Supplement: Supplementary file 1 — Supplementary Information. [file 41598_2020_79657_MOESM1_ESM.docx]

**Epidural analgesia does not impact recurrence or mortality in patients after rectal cancer resection**

Hsiang-Ling Wu^1,2^, Ying-Hsuan Tai^1,2,3,4^, Shih-Pin Lin^1,2^, Shung-Haur Yang^2,5,6^, Mei-Yung Tsou^1,2^, Kuang-Yi Chang^1,2,*^

^1^ Department of Anaesthesiology, Taipei Veterans General Hospital, Taipei, Taiwan

^2^ School of Medicine, National Yang-Ming University, Taipei, Taiwan

^3^ Department of Anaesthesiology, Shuang Ho Hospital, Taipei Medical University, New Taipei City, Taiwan

^4^ Department of Anaesthesiology, School of Medicine, College of Medicine, Taipei Medical University, Taipei, Taiwan

^5^ Division of Colon and Rectal Surgery, Department of Surgery, Taipei Veterans General Hospital, Taipei, Taiwan

^6^ National Yang-Ming University Hospital, Yilan, Taiwan

*** Corresponding author:** Dr. Chang: [kychang@vghtpe.gov.tw](mailto:kychang@vghtpe.gov.tw)

Department of Anaesthesiology, Taipei Veterans General Hospital, No. 201, Sec. 2, Shih-pai Rd., Taipei 11217, Taiwan. Tel: +886-2-28757549; Fax: +886-2-28751597

Supplementary Table S1: The result of logistic regression model for inverse probability of treatment weighting and quintile stratification analysis

|  | **OR (95% CI)** | ***p*** |
| --- | --- | --- |
| **Age** | 1.015 (1.001 – 1.030) | 0.038 |
| **Sex, M vs. F** | 1.147 (0.829 – 1.587) | 0.406 |
| **ASA class ≥ 3** | 0.874 (0.578 – 1.323) | 0.525 |
| **Diabetes** | 1.173 (0.785 – 1.754) | 0.435 |
| **Coronary arterial disease** | 1.027 (0.582 – 1.812) | 0.928 |
| **Heart failure** | 0.502 (0.227 – 1.112) | 0.090 |
| **Stroke** | 0.870 (0.435 – 1.737) | 0.692 |
| **Chronic kidney disease** | 1.327 (0.820 – 2.149) | 0.249 |
| **Pretreatment CEA^†^** | 1.001 (0.666 – 1.504) | 0.998 |
| **Anaesthesia time^‡^** | 0.312 (0.210 – 0.464) | <.001 |
| **Laparoscopic surgery** | 0.155 (0.037 – 0.642) | 0.010 |
| **pRBC transfusion** | 1.071 (0.709 – 1.618) | 0.743 |
| **Stage** |  | 0.052 |
| II vs. I | 1.214 (0.794 – 1.858) | 0.371 |
| III vs. I | 1.892 (1.117 – 3.205) | 0.018 |
| **Distance from anal verge, cm^‡^** | 0.973 (0.929 – 1.019) | 0.241 |
| **Tumour differentiation** |  | 0.272 |
| Moderate vs. good | 0.647 (0.356 – 1.178) | 0.155 |
| Poor vs. good | 0.929 (0.329 – 2.624) | 0.889 |
| **Mucinous histology** | 1.056 (0.385 – 2.898) | 0.916 |
| **Signet-ring histology** | 0.528 (0.129 – 2.163) | 0.374 |
| **Lymphovascular invasion** | 0.771 (0.467 – 1.273) | 0.309 |
| **Perineural invasion** | 0.610 (0.280 – 1.328) | 0.213 |
| **Postoperative C/T** | 0.622 (0.401 – 0.964) | 0.034 |
| **Postoperative R/T** | 0.888 (0.378 – 2.087) | 0.785 |
| **Preoperative C/T ± R/T** | 0.894 (0.586 – 1.364) | 0.604 |

OR: odds ratio; CI: confidence interval; M: male, F: female; ASA: American Society of Anesthesiologists; CEA: carcinoembryonic antigen; C/T: chemotherapy; pRBC: packed red blood cells; R/T: radiotherapy. †On base-10 logarithmic scale; ‡On base-2 logarithmic scale
